# Adulterations in Medicine

**Published:** 1847-08

**Authors:** 


### ADULTERATIONS IN MEDICINE.

Prof. Lewis C. Beck has published a volume of upwards of three hundred pages on this subject, intended as a manual for the physician, the apothecary, and the artisan. He exhibits the adulterations to which the various substances used in medicine are subject, and describes the processes by which they may be detected. The processes given are for the most part so simple that the tyro in chemistry may apply them; but in every case this is not possible, and in casting his eye over these pages the student will be impressed with the importance of acquiring a somewhat more perfect knowledge of chemical science than falls to the lot of too many students of medicine. The utility of a work of this character will appear to every one, and it will be admitted by his brethren that the task of preparing it could not have fallen to abler hands than Dr. Beck's.

